# 5-(2,4-Dichloro­phen­oxy)-1,3-dimethyl-1*H*-pyrazole-4-carbaldehyde

**DOI:** 10.1107/S1600536811045831

**Published:** 2011-11-05

**Authors:** Yong-Jun Shen, Mei Xu, Chong-Guang Fan

**Affiliations:** a College of Chemistry and Chemical Engineering, Nantong University, Nantong 226019, People’s Republic of China; b Department of Chemistry and Environmental Science, Cangzhou Normal University, Cangzhou 061001, People’s Republic of China

## Abstract

In the title mol­ecule, C_12_H_10_Cl_2_N_2_O_2_, the benzene and pyrazole rings form a dihedral angle of 72.8 (3)°. In the crystal, weak inter­molecular C—H⋯O hydrogen bonds link the mol­ecules into chains along [01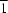].

## Related literature

For the crystal structure of a related pyrazole derivative studied recently by our group, see: Shen *et al.* (2011 ▶).
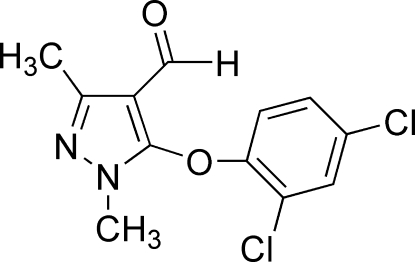


## Experimental

### 

#### Crystal data

C_12_H_10_Cl_2_N_2_O_2_
*M* *_r_* = 285.12
Triclinic, 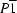

*a* = 8.018 (3) Å
*b* = 8.505 (3) Å
*c* = 10.365 (5) Å
α = 74.56 (2)°
β = 83.96 (3)°
γ = 67.30 (2)°
*V* = 628.5 (5) Å^3^
*Z* = 2
Mo *K*α radiation
μ = 0.51 mm^−1^
*T* = 113 K
0.24 × 0.22 × 0.20 mm

#### Data collection

Rigaku Saturn724 CCD diffractometer
Absorption correction: multi-scan (*CrystalClear*; Rigaku, 2008 ▶) *T*_min_ = 0.887, *T*_max_ = 0.905
6391 measured reflections
2948 independent reflections
2766 reflections with *I* > 2σ(*I*)
*R*_int_ = 0.059

#### Refinement

*R*[*F* ^2^ > 2σ(*F* ^2^)] = 0.054
*wR*(*F* ^2^) = 0.146
*S* = 1.06
2948 reflections
165 parameters
H-atom parameters constrained
Δρ_max_ = 0.40 e Å^−3^
Δρ_min_ = −0.59 e Å^−3^

### 

Data collection: *CrystalClear* (Rigaku, 2008 ▶); cell refinement: *CrystalClear*; data reduction: *CrystalClear*; program(s) used to solve structure: *SHELXS97* (Sheldrick, 2008 ▶); program(s) used to refine structure: *SHELXL97* (Sheldrick, 2008 ▶); molecular graphics: *SHELXTL* (Sheldrick, 2008 ▶); software used to prepare material for publication: *SHELXTL*.

## Supplementary Material

Crystal structure: contains datablock(s) global, I. DOI: 10.1107/S1600536811045831/cv5188sup1.cif

Structure factors: contains datablock(s) I. DOI: 10.1107/S1600536811045831/cv5188Isup2.hkl

Supplementary material file. DOI: 10.1107/S1600536811045831/cv5188Isup3.cml

Additional supplementary materials:  crystallographic information; 3D view; checkCIF report

## Figures and Tables

**Table 1:** Hydrogen-bond geometry (Å, °)

*D*—H⋯*A*	*D*—H	H⋯*A*	*D*⋯*A*	*D*—H⋯*A*
C4—H4⋯O2^i^	0.95	2.45	3.284 (3)	147
C10—H10*C*⋯O2^ii^	0.98	2.46	3.391 (3)	158

Symmetry codes: (i) 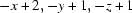; (ii) 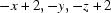.

**Fig. 1.**
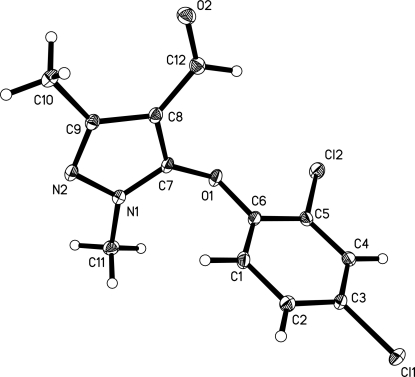
The molecular structure of (I), with displacement ellipsoids drawn at the 30% probability level.

## supplementary crystallographic information

### Comment

As a continuation of our structural study of pyrazole derivatives (Shen *et al.*, 2011), we present here the title compound (I).

In (I) (Fig. 1), all bond lengths and angles are normal and correspond to those observed in the related compound (Shen *et al.*, 2011). The dihedral angle between the substituted benzene ring and pyrazole ring is 72.8 (3) °. The crystal packing exhibits weak intermolecular C—H···O interactions (Table 1), which link molecules  into chains along [01-1].

### Experimental

To a stirred solution of 1-methyl-3-methyl-5-chloro-1*H*-pyrazole- 4-carbaldehyde(30 mmol) and 2,4-dichlorophenol(48 mmol) in DMF(30 ml) was added potassium hydroxide(60 mmol) at room temperature. The resulting mixture was heated to 388 k for 6 h. Then the reaction solution was poured into cold water(100 ml) and extracted with ethyl acetate (3 * 60 ml). The organic layer was dried over anhydrous magnesium sulfate. After removal of the solvent, the residue was recrystallized from ethyl acetate/petroleum ether to give colourless crystals.

### Refinement

All H atoms were placed in calculated positions, with C–H = 0.95 - 0.98 ° A, and included in the final cycles of refinement using a riding model, with *U*_iso_(H) = 1.2-1.5 *U*_eq_(C).

### Crystal data

**Table:**

C_12_H_10_Cl_2_N_2_O_2_	*Z* = 2
*M**_r_* = 285.12	*F*(000) = 292
Triclinic, *P*1	*D*_x_ = 1.507 Mg m^−^^3^
Hall symbol: -P 1	Mo *K*α radiation, λ = 0.71073 Å
*a* = 8.018 (3) Å	Cell parameters from 2204 reflections
*b* = 8.505 (3) Å	θ = 2.0–27.9°
*c* = 10.365 (5) Å	µ = 0.51 mm^−^^1^
α = 74.56 (2)°	*T* = 113 K
β = 83.96 (3)°	Prism, colourless
γ = 67.30 (2)°	0.24 × 0.22 × 0.20 mm
*V* = 628.5 (5) Å^3^	

### Data collection

**Table:**

Rigaku Saturn724 CCD diffractometer	2948 independent reflections
Radiation source: rotating anode	2766 reflections with *I* > 2σ(*I*)
multilayer	*R*_int_ = 0.059
Detector resolution: 14.22 pixels mm^-1^	θ_max_ = 27.9°, θ_min_ = 2.0°
ω and φ scans	*h* = −10→9
Absorption correction: multi-scan (*CrystalClear*; Rigaku, 2008)	*k* = −11→11
*T*_min_ = 0.887, *T*_max_ = 0.905	*l* = −13→13
6391 measured reflections	

### Refinement

**Table:**

Refinement on *F*^2^	Primary atom site location: structure-invariant direct methods
Least-squares matrix: full	Secondary atom site location: difference Fourier map
*R*[*F*^2^ > 2σ(*F*^2^)] = 0.054	Hydrogen site location: inferred from neighbouring sites
*wR*(*F*^2^) = 0.146	H-atom parameters constrained
*S* = 1.06	*w* = 1/[σ^2^(*F*_o_^2^) + (0.0863*P*)^2^ + 0.4717*P*] where *P* = (*F*_o_^2^ + 2*F*_c_^2^)/3
2948 reflections	(Δ/σ)_max_ = 0.002
165 parameters	Δρ_max_ = 0.40 e Å^−^^3^
0 restraints	Δρ_min_ = −0.59 e Å^−^^3^

### Special details

**Table:**

Geometry. All e.s.d.'s (except the e.s.d. in the dihedral angle between two l.s. planes) are estimated using the full covariance matrix. The cell e.s.d.'s are taken into account individually in the estimation of e.s.d.'s in distances, angles and torsion angles; correlations between e.s.d.'s in cell parameters are only used when they are defined by crystal symmetry. An approximate (isotropic) treatment of cell e.s.d.'s is used for estimating e.s.d.'s involving l.s. planes.
Refinement. Refinement of *F*^2^ against ALL reflections. The weighted *R*-factor *wR* and goodness of fit *S* are based on *F*^2^, conventional *R*-factors *R* are based on *F*, with *F* set to zero for negative *F*^2^. The threshold expression of *F*^2^ > σ(*F*^2^) is used only for calculating *R*-factors(gt) *etc*. and is not relevant to the choice of reflections for refinement. *R*-factors based on *F*^2^ are statistically about twice as large as those based on *F*, and *R*- factors based on ALL data will be even larger.

### Fractional atomic coordinates and isotropic or equivalent isotropic displacement parameters (Å^2^)

**Table:**

	*x*	*y*	*z*	*U*_iso_*/*U*_eq_	
Cl1	0.32121 (7)	0.81388 (7)	0.29679 (5)	0.03004 (17)	
Cl2	0.90839 (7)	0.84252 (7)	0.48228 (5)	0.02853 (17)	
O1	0.7986 (2)	0.68007 (19)	0.73949 (13)	0.0229 (3)	
O2	1.1787 (2)	0.15447 (19)	0.88478 (15)	0.0268 (3)	
N1	0.6770 (2)	0.6174 (2)	0.95613 (15)	0.0200 (3)	
N2	0.7105 (2)	0.4841 (2)	1.07097 (15)	0.0203 (3)	
C1	0.5338 (3)	0.6535 (3)	0.66259 (18)	0.0220 (4)	
H1	0.5090	0.5963	0.7502	0.026*	
C2	0.4222 (3)	0.6874 (3)	0.55656 (19)	0.0233 (4)	
H2	0.3203	0.6540	0.5711	0.028*	
C3	0.4611 (3)	0.7702 (3)	0.42976 (19)	0.0225 (4)	
C4	0.6096 (3)	0.8200 (2)	0.40452 (19)	0.0220 (4)	
H4	0.6347	0.8762	0.3167	0.026*	
C5	0.7201 (3)	0.7854 (2)	0.51096 (18)	0.0201 (4)	
C6	0.6823 (3)	0.7040 (2)	0.63958 (18)	0.0194 (4)	
C7	0.7948 (3)	0.5673 (2)	0.85978 (17)	0.0186 (4)	
C8	0.9135 (3)	0.3965 (2)	0.90937 (18)	0.0194 (4)	
C9	0.8528 (3)	0.3516 (2)	1.04350 (18)	0.0188 (4)	
C10	0.9292 (3)	0.1822 (3)	1.1457 (2)	0.0249 (4)	
H10A	0.8626	0.1912	1.2300	0.037*	
H10B	1.0569	0.1563	1.1602	0.037*	
H10C	0.9185	0.0875	1.1143	0.037*	
C11	0.5330 (3)	0.7878 (3)	0.9522 (2)	0.0245 (4)	
H11A	0.5564	0.8774	0.8794	0.037*	
H11B	0.5286	0.8182	1.0376	0.037*	
H11C	0.4170	0.7822	0.9369	0.037*	
C12	1.0679 (3)	0.3008 (3)	0.8386 (2)	0.0226 (4)	
H12	1.0848	0.3566	0.7485	0.027*	

### Atomic displacement parameters (Å^2^)

**Table:**

	*U*^11^	*U*^22^	*U*^33^	*U*^12^	*U*^13^	*U*^23^
Cl1	0.0258 (3)	0.0415 (3)	0.0194 (3)	−0.0095 (2)	−0.00688 (19)	−0.0039 (2)
Cl2	0.0265 (3)	0.0386 (3)	0.0221 (3)	−0.0183 (2)	0.00085 (19)	−0.0012 (2)
O1	0.0273 (7)	0.0298 (7)	0.0143 (6)	−0.0164 (6)	−0.0027 (5)	0.0001 (5)
O2	0.0248 (7)	0.0256 (7)	0.0293 (7)	−0.0064 (6)	0.0004 (6)	−0.0104 (6)
N1	0.0201 (8)	0.0219 (8)	0.0159 (7)	−0.0060 (6)	−0.0012 (6)	−0.0034 (6)
N2	0.0240 (8)	0.0236 (8)	0.0142 (7)	−0.0107 (7)	−0.0025 (6)	−0.0022 (6)
C1	0.0255 (10)	0.0260 (9)	0.0141 (8)	−0.0108 (8)	0.0007 (7)	−0.0030 (7)
C2	0.0218 (9)	0.0284 (10)	0.0206 (9)	−0.0108 (8)	−0.0001 (7)	−0.0051 (7)
C3	0.0226 (9)	0.0262 (9)	0.0149 (8)	−0.0045 (8)	−0.0027 (7)	−0.0049 (7)
C4	0.0207 (9)	0.0216 (9)	0.0175 (8)	−0.0027 (7)	0.0014 (7)	−0.0032 (7)
C5	0.0199 (9)	0.0215 (9)	0.0169 (8)	−0.0069 (7)	0.0029 (7)	−0.0039 (7)
C6	0.0207 (9)	0.0202 (8)	0.0151 (8)	−0.0048 (7)	−0.0024 (7)	−0.0041 (6)
C7	0.0218 (9)	0.0231 (9)	0.0131 (7)	−0.0110 (7)	−0.0026 (6)	−0.0030 (6)
C8	0.0204 (9)	0.0232 (9)	0.0165 (8)	−0.0100 (7)	−0.0016 (7)	−0.0049 (7)
C9	0.0207 (9)	0.0210 (8)	0.0165 (8)	−0.0094 (7)	−0.0026 (6)	−0.0041 (6)
C10	0.0272 (10)	0.0230 (9)	0.0216 (9)	−0.0087 (8)	−0.0062 (7)	0.0005 (7)
C11	0.0232 (10)	0.0215 (9)	0.0254 (9)	−0.0038 (8)	−0.0032 (7)	−0.0061 (7)
C12	0.0235 (9)	0.0251 (9)	0.0222 (9)	−0.0101 (8)	0.0001 (7)	−0.0092 (7)

### Geometric parameters (Å, °)

**Table:**

Cl1—C3	1.740 (2)	C4—C5	1.386 (3)
Cl2—C5	1.731 (2)	C4—H4	0.9500
O1—C7	1.361 (2)	C5—C6	1.391 (3)
O1—C6	1.388 (2)	C7—C8	1.385 (3)
O2—C12	1.219 (3)	C8—C9	1.426 (3)
N1—C7	1.338 (2)	C8—C12	1.443 (3)
N1—N2	1.374 (2)	C9—C10	1.489 (3)
N1—C11	1.456 (3)	C10—H10A	0.9800
N2—C9	1.328 (3)	C10—H10B	0.9800
C1—C2	1.390 (3)	C10—H10C	0.9800
C1—C6	1.392 (3)	C11—H11A	0.9800
C1—H1	0.9500	C11—H11B	0.9800
C2—C3	1.382 (3)	C11—H11C	0.9800
C2—H2	0.9500	C12—H12	0.9500
C3—C4	1.389 (3)		
			
C7—O1—C6	118.55 (15)	N1—C7—C8	108.81 (16)
C7—N1—N2	110.80 (16)	O1—C7—C8	128.76 (17)
C7—N1—C11	128.50 (17)	C7—C8—C9	103.53 (16)
N2—N1—C11	120.66 (16)	C7—C8—C12	125.35 (17)
C9—N2—N1	105.68 (15)	C9—C8—C12	130.95 (18)
C2—C1—C6	119.56 (17)	N2—C9—C8	111.17 (17)
C2—C1—H1	120.2	N2—C9—C10	121.08 (17)
C6—C1—H1	120.2	C8—C9—C10	127.74 (18)
C3—C2—C1	119.33 (19)	C9—C10—H10A	109.5
C3—C2—H2	120.3	C9—C10—H10B	109.5
C1—C2—H2	120.3	H10A—C10—H10B	109.5
C2—C3—C4	121.98 (18)	C9—C10—H10C	109.5
C2—C3—Cl1	119.51 (16)	H10A—C10—H10C	109.5
C4—C3—Cl1	118.50 (15)	H10B—C10—H10C	109.5
C5—C4—C3	118.21 (17)	N1—C11—H11A	109.5
C5—C4—H4	120.9	N1—C11—H11B	109.5
C3—C4—H4	120.9	H11A—C11—H11B	109.5
C4—C5—C6	120.74 (18)	N1—C11—H11C	109.5
C4—C5—Cl2	119.32 (14)	H11A—C11—H11C	109.5
C6—C5—Cl2	119.94 (15)	H11B—C11—H11C	109.5
O1—C6—C5	116.08 (17)	O2—C12—C8	125.49 (19)
O1—C6—C1	123.75 (16)	O2—C12—H12	117.3
C5—C6—C1	120.17 (17)	C8—C12—H12	117.3
N1—C7—O1	122.15 (17)		
			
C7—N1—N2—C9	0.7 (2)	C11—N1—C7—O1	2.7 (3)
C11—N1—N2—C9	−177.30 (16)	N2—N1—C7—C8	−0.6 (2)
C6—C1—C2—C3	0.2 (3)	C11—N1—C7—C8	177.13 (18)
C1—C2—C3—C4	0.4 (3)	C6—O1—C7—N1	−85.0 (2)
C1—C2—C3—Cl1	−179.69 (15)	C6—O1—C7—C8	101.7 (2)
C2—C3—C4—C5	−0.3 (3)	N1—C7—C8—C9	0.3 (2)
Cl1—C3—C4—C5	179.75 (14)	O1—C7—C8—C9	174.29 (17)
C3—C4—C5—C6	−0.4 (3)	N1—C7—C8—C12	−175.30 (17)
C3—C4—C5—Cl2	178.92 (14)	O1—C7—C8—C12	−1.3 (3)
C7—O1—C6—C5	−165.15 (17)	N1—N2—C9—C8	−0.4 (2)
C7—O1—C6—C1	15.6 (3)	N1—N2—C9—C10	179.92 (16)
C4—C5—C6—O1	−178.22 (16)	C7—C8—C9—N2	0.1 (2)
Cl2—C5—C6—O1	2.5 (2)	C12—C8—C9—N2	175.37 (19)
C4—C5—C6—C1	1.0 (3)	C7—C8—C9—C10	179.69 (18)
Cl2—C5—C6—C1	−178.27 (15)	C12—C8—C9—C10	−5.0 (3)
C2—C1—C6—O1	178.23 (17)	C7—C8—C12—O2	175.37 (19)
C2—C1—C6—C5	−0.9 (3)	C9—C8—C12—O2	1.0 (3)
N2—N1—C7—O1	−175.06 (16)		

### Hydrogen-bond geometry (Å, °)

**Table:**

*D*—H···*A*	*D*—H	H···*A*	*D*···*A*	*D*—H···*A*
C4—H4···O2^i^	0.95	2.45	3.284 (3)	147
C10—H10C···O2^ii^	0.98	2.46	3.391 (3)	158

Symmetry codes: (i) −*x*+2, −*y*+1, −*z*+1; (ii) −*x*+2, −*y*, −*z*+2.
